# Exposure and risk assessment of urinary trans, trans-Muconic acid in school-age children in the vicinity of a petrochemical complex in Central Taiwan

**DOI:** 10.3389/fpubh.2023.1234823

**Published:** 2023-09-04

**Authors:** Po-Keng Cheng, Vinoth Kumar Ponnusamy, Karthikeyan Prakasham, Hsin-I Huang, Wan-Ting Chang, Po-Chin Huang

**Affiliations:** ^1^Department of Finance and Cooperative Management, National Taipei University, New Taipei City, Taiwan; ^2^Department of Medicinal and Applied Chemistry, Kaohsiung Medical University, Kaohsiung City, Taiwan; ^3^Research Center for Precision Environmental Medicine, and Ph.D. Program in Environmental and Occupational Medicine, College of Medicine, Kaohsiung Medical University, Kaohsiung City, Taiwan; ^4^Department of Chemistry, National Sun Yat-Sen University, Kaohsiung City, Taiwan; ^5^Department of Medical Research, Kaohsiung Medical University Hospital (KMUH), Kaohsiung City, Taiwan; ^6^National Institute of Environmental Health Sciences, National Health Research Institutes, Miaoli, Taiwan; ^7^Department of Medical Research, China Medical University Hospital, China Medical University, Taichung, Taiwan

**Keywords:** benzene, children, petrochemical complex, trans, trans-muconic acid, urine

## Abstract

School-age children living near large petrochemical factories may be at high risk of exposure to benzene released during manufacturing processes. We aimed to investigate the urinary concentrations of trans, trans-muconic acid (t,t-MA) in school-age children living near a petrochemical complex and to estimate their cumulative risk of benzene exposure. We examined an established cohort (Taiwan Petrochemical Complex Cohort for Children, TPE3C) of school-age children (aged 6–13 years) who lived near large petrochemical factories in central Taiwan between October 2013 and September 2014. The cohort comprised 297 children from five elementary schools, namely S.-C. Branch (n = 63, school A, ~0.9 km), F.-A. (*n* = 51, school B, ~2.7 km), C.-T. (*n* = 63, school C, ~5.5 km), M.-L. (*n* = 54, school D, ~6.9 km), and L.-F. (*n* = 66, school E, ~8.6 km). We analyzed the urinary t,t-MA levels of each participant and estimated their daily intake of benzene. We also performed multiple regression analysis to investigate potential risk factors for a high urinary t,t-MA level in the study cohort. The median urinary t,t-MA levels and median estimated benzene daily intake of the children from each school were as follows: school A, 64.07 ng/mL, 11.13 μg/kg/day; school B, 61.01 ng/mL, 15.32 μg/kg/day; school C, 59.38 ng/mL, 14.81 μg/kg/day; school D, 42.35 ng/mL, 11.67 μg/kg/day; school E, undetected, 0.14 μg/kg/day. The distance between a school and a petrochemical complex (greater distance: β = −0.26, 95% confidence interval [CI] = −0.52 to 0.00, *p* = 0.053), and the age of the children (older age: β = −3.44, 95% CI = −5.90 to −1.46, *p* < 0.001) were identified as potential risk factors. After confounders were adjusted for, the creatinine adjusted urinary t,t-MA levels of the school-age children tended to be lower when the distance between their school and a petrochemical complex was greater.

## 1. Introduction

Benzene (molecular formula, C_6_H_6_) is a colorless, highly flammable, and toxic chemical compound. It forms naturally in crude oil and coal tar, and it is also a byproduct of incomplete organic matter combustion. Benzene is used in various industrial processes, such as the production of lubricants, plastics, rubber products, dyes, and synthetic fibers. Additionally, benzene is present in substances such as gasoline, paints, solvents, and cigarette smoke, and it is released into the environment through industrial activities such as oil refinement and manufacturing.

Benzene is widely recognized as a human carcinogen that can lead to various health conditions, including leukemia, anemia, and central nervous system damage. International agencies have classified benzene as a Group 1 human carcinogen (IARC) because it is associated with an increased risk of leukemia and blood disorders such as aplastic anemia or myelodysplastic syndrome among individuals with high exposure to benzene [(1, 2)]. Furthermore, acute symptoms associated with high occupational exposure to benzene include dizziness, headache, and nausea (3).

The main sources of benzene exposure are occupational exposure, environmental exposure, and tobacco smoke. Exposure to benzene can occur through inhalation, ingestion, or skin contact, with inhalation being the most common route (4, 5). Benzene in air may originate from various sources, including motor vehicle exhaust, industrial emissions, tobacco smoking, gasoline vapor, or the burning of coal, oil, or wood (6). People can also be exposed to benzene through ingestion, such as by drinking water contaminated with benzene. Additionally, benzene can be absorbed through the skin, although this is an uncommon route of exposure.

Accurately determining the dosage absorbed by a benzene-exposed population is crucial for accurately assessing the health effects of benzene exposure. After benzene is absorbed, it is metabolized in the body to form benzene oxide, which is then converted in the liver and lungs to either trans, trans-muconic acid (t,t-MA) or S-phenylmercapturic acid (SPMA) (7). Because the most frequently used biomarkers for determining occupational benzene exposure are t,t-MA and SPMA (8–10), we used t,t-MA and SPMA as the biomarkers for assessing benzene exposure in the present study.

Few studies have investigated specific populations who are susceptible to benzene exposure, especially children. Individuals who live near to or downwind from production facilities may be exposed to atmospheric levels of benzene that are higher than the ambient background level. Therefore, the objective of the present study was to use t,t-MA and SPMA as biomarkers to investigate whether the benzene exposure of elementary school students decreases when their elementary school was located farther away from a nearby petrochemical complex.

## 2. Materials and methods

### 2.1. Ethics statement

The Institutional Review Board of the National Health Research Institutes approved the study protocol (Nos. EC1020607 and EC1110122) of the present study. Prior to study enrollment, informed consent was obtained from all child participants and their parents.

### 2.2. Participants and study design

**Figure 1:**
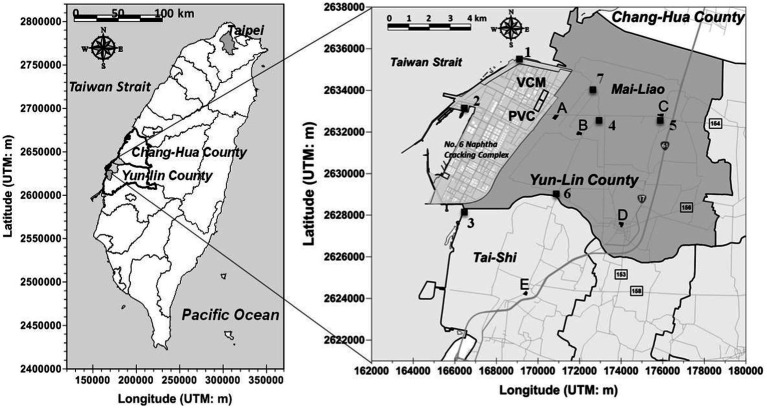
Locations of air VOCs monitoring sites insides (1 ~ 3) and outsides (4 ~ 7) a petrochemical complex (No. 6 Naphtha Cracking Complex) in central Taiwan. The air monitoring data was measured by local EPA of Yun-Lin County, Taiwan during May 2012 to June 2014.

We examined a well-established cohort (Taiwan Petrochemical Complex Cohort for Children, TPE3C) of school-age children (6–13 years old) who lived close to vinyl chloride monomer (VCM) and polyvinyl chloride (PVC) factories located within a petrochemical complex in Yunlin County, Taiwan, between October 2013 and September 2014 [(11–13)]. In total, 344 children were enrolled from five elementary schools: *Syu-Cuo* Branch (*n* = 69, school A, ~0.9 km), *Feng-An* (*n* = 59, school B, ~2.7 km), *Ciao-Tou* (*n* = 67, school C, ~5.5 km), *Mai-Liao* (*n* = 75, school D, ~6.9 km), and *Lung-Feng* (*n* = 74, school E, ~8.6 km) (see Figure 1). Each student was randomly matched by gender on the basis of their school identification number. A student was eligible for inclusion if they were in grade 1–6 of elementary school, had been living at their reported place of residence for at least 1 year, and were aged ≥6 years. In accordance with the guidelines of the World Health Organization (WHO), we excluded children who had urinary creatinine concentrations of <30 mg/dL or > 300 mg/dL as determined through valid urine samples [*n* = 20; (14)]. Children who consumed vitamin B complexes less than 1 week before the start of the study (*n* = 5) or those who had chronic hepatitis B or C (*n* = 1) infection were excluded. We also excluded children with insufficient biochemistry data (*n* = 21). After the aforementioned inclusion and exclusion criteria were applied, 297 children were enrolled as participants in the present study.

### 2.3. Sample collection

Our sampling occurred between October 2013 and September 2014. We have described the details in our previous published articles (11–13, 15). Briefly, the sampling dates for each school were as follows: A (October 16, 2013), B (November 16, 2013), C (March 5, 2014), D (April 9, 2014), and E (September 24, 2014). On Wednesday morning, each participant had their first-morning urine sample (< 60 mL) collected using a plastic urine bag (PP), which was then immediately transferred into an amber glass bottle and stored at −80°C.

### 2.4. Analytical method for trans, trans-muconic acid

We employed an established method (16) to analyze all samples. In brief, analytical grade standard analytes, such as SPMA, D_5_-S-phenylmercapturic acid, and D_4_-t,t-Muconic acid, were purchased from Toronto Research Chemicals, Canada, and Thiodiglycolic acid and t,t-muconic acid (TTMA) were purchased from Alfa Aesar (MA, United States). Ultra-high liquid chromatography with tandem mass spectrometry was performed using a Shimadzu UHPLC–MS/MS system (Shimadzu, Kyoto, Japan) with a low-pressure gradient mixer that was equipped with a quaternary pump (Model: Nexera I 2040) and had an autosampler capacity of 20 μL (injector loop). The mass spectrometer was coupled with a mixed-mode ESI source (8,045 LCMS). Liquid chromatography–mass spectrometry and the processing of results were performed using LabSolutions software (Version 2.6). Chromatographic separation was achieved at 35°C on a C18 column (150 mm × 4.6 mm I.D.; particle size, 2.7 μm; sun shell brand) in an oven. Chromatographic analysis was conducted in gradient mode and initiated with a mobile phase at an 80:20 concentration ratio (A [0.1% formic acid in water] to B [methanol]) for 3.5 min. During the second step, the ratio was changed to 35:65 and kept constant for approximately 4.0 min. During the third step, the ratio was changed to 25:75 over 5 min and kept constant for 6 min (6.1 to 9 min). For the entirety of the analysis process, the flow rate was set to 0.4 mL min^−1^ with a pretreated-sample injection volume of 3 μL. To measure five internal standards and their corresponding internal standards, ESI-MS/MS parameters were optimized in a negative multiple-reaction monitoring mode. A novel fast-urinary-metabolites-extraction technique was employed to perform urine sample pretreatment by using two syringes in tandem in a liquid–liquid microextraction (LLME) and micro solid-phase extraction (μSPE) setup. Subsequently, 0.5 mL of urine sample was spiked with 20 μL of internal standard solution; 1 mL acidified methanol (using 0.1% formic acid pH 3) was then added as an extraction solvent in the LLME extraction syringe setup, and the resulting mixture was vortexed for 2 min. During the second step, the LLME setup was connected to a μSPE syringe, which was prepacked with a top layer comprising 500-mg anhydrous MgSO_4_ between the two frits (top and the middle). In the second layer, 50 mg of C18 and SiO_2_ were added between the middle and bottom frits. During the third step, the LLME and μSPE setups were placed in an auto-plunging device, the extractant was allowed to pass through the μSPE syringe column at a flow speed of 0.25 mL/min, and the cleaned extractants were collected in a 10-mL glass tube. During the fourth step, the collected extractants obtained after the clean-up process were dried using a high-pressure parallel vacuum evaporator (HPPE-40, GE Tech, Kaohsiung, Taiwan) at 35°C. Finally, the residues were dissolved in 100 μL of methanol for the subsequent UHPLC–MS/MS analysis. The recovery and relative difference percentage of the spiked (1, 5, and 20 ng/mL) and repeated (precision) sample was defined as within ±15% and ± 30%, respectively, for each batch. For low concentration t,t-MA, the quality control accuracy was between 97.9 and 108.0%; and for high concentration t,t-MA, the quality control accuracy was between 96.2 and 107.6%.

### 2.5. Estimation of daily benzene intake and hazard quotients

We estimated the daily intake (DI) of benzene on the basis of urinary t,t-MA levels. In the formula for calculating the DI of benzene [equation 1; (17–19)], *UE* is the urinary excretion of the measured urinary metabolites per gram of creatinine; *CE_smoothed_* is the smoothed creatinine excretion rate, which is based on participants’ age, body weight (*BW*) and height (*ht*) as well as gender-based values associated with the rate of urinary creatinine excretion (20, 21); *F_UE_* is the fractional urinary excretion of benzene [i.e., t,t-MA (3.9%)] levels in humans (18, 22, 23); *MW_d_* is the molar weight of benzene (78.1); and *MW_m_* is the molar weight of t,t-MA (142.1).

(1) $$\begin{array}{l} \mathrm{Daily}\;\mathrm{intake}\;\left(\mathrm{\mu g}/\mathrm{kg}/\mathrm{day}\right)=\\ \;\frac{UE\left(\mathrm{\mu g}/g\;\mathrm{crea}\right)\times CE_{\mathrm{smoothed}}\left(\mathrm{mg}/\mathrm{day}\right)}{F_{UE}\times BW\left(\mathrm{kg}\right)\times 1000\left(\mathrm{mg}/g\right)}\times \frac{MW_{d}}{MW_{m}} \end{array}$$

$$For\;\mathrm{minors}\;\left(\geq 6-<18\;\mathrm{years}\;\mathrm{old}\right):$$

$$\begin{array}{l} CE_{\mathrm{smoothed}}=ht\times \left\{6.265+0.0564\times \left(ht-168\right)\right\}\ldots \\ \;ht<168\;\mathrm{cm}\ldots \left(\mathrm{male}\right) \end{array}$$

$$\begin{array}{l} CE_{\mathrm{smoothed}}=ht\times \left\{6.265+0.2550\times \left(ht-168\right)\right\}\ldots \\ \;ht\geq 168\;\mathrm{cm}\ldots \left(\mathrm{male}\right) \end{array}$$

$$CE_{\mathrm{smoothed}}=2.045\times ht\times \exp\left\{0.01552\times \left(ht-90\right)\right\}\ldots \left(\mathrm{female}\right)$$

We utilized the hazard quotient (HQ) to calculate each participant’s risk from benzene. The formula for HQ is as follows (17). Reference doses (RfDs) of benzene by the US Environmental Protection Agency (US EPA) is 4 μg/kg/day, by Agency for Toxic Substances and Disease Registry (ATSDR) is 0.5 μg/kg/day, and by Firth (24) is 400 μg/kg/day.

(2) $$HQ=\frac{DI}{\mathrm{Reference}\;\mathrm{dose}}$$

### 2.6. Statistical analysis

We conducted a Kruskal–Wallis test to compare the urinary t,t-MA and benzene DI levels of the participants from the five elementary schools. We also conducted analysis of covariance (ANCOVA, after adjustment for confounders) and the Jonckheere–Terpstra test to identify the differences in the urinary t,t-MA and benzene DI levels of the participants. In addition, we compared the urinary t,t-MA and SPMA levels of the children in relation to several factors (e.g., passive smoking, home close to main road, and factories within 1 km near living environment) by Mann–Whitney U test. Multiple regression analysis was performed to assess the association between the creatinine-adjusted urinary t,t-MA (μg/g creatinine) levels of the participants and the location of their elementary schools after adjustments were made for several covariates (e.g., age, gender, passive smoking, close proximity of a person’s home to a main road). Creatinine adjusted urinary t,t-MA levels of the participants were natural logarithm transformed to approximate normal distribution. We used school A as a reference and the order of other schools were increased by the increasing distance to the source. We used a girl, non-smoker, never served in petrochemical complex, and home not close to main road as a reference of gender, passive smoking, father’s job, and home close to main road, respectively. We applied stepwise regression in our multiple regression analysis. We also conducted sensitivity analysis using only the children who were not passively exposed to smoking or their fathers were not employed at the petrochemical complex to assess the robustness of the reported associations. All statistical analyses were conducted using R version 4.1.2 (R Foundation for Statistical Computing, Vienna, Austria).

## 3. Results

### 3.1. Demographic characteristics of participants

Table 1 lists the demographic characteristics of the study participants. Their mean age and body mass index (BMI) score were 9.8 ± 1.7 years (range, 6.1–12.5 years) and 19.0 ± 4.1 kg/m^2^ (range, 12.2–32.7 kg/m^2^), respectively. The participants were evenly balanced in terms of gender (boys, 50.2%), and approximately half of the study participants were from households with annual income of less than US$15,600 (50.5%). Nearly half of the study participants’ fathers were working at a petrochemical complex (48.8%), and approximately 20% of their mothers were working at a petrochemical complex (19.5%). Approximately 80% of the study participants reported a mild-to-moderate physical activity level (80.5%), and 65% of them were exposed to passive smoking.

**Table 1:** Sociodemographic characteristics of the participating children.

Characteristics	Total (*N* = 297)
*Continuous variable, mean ± SD (range)*	
Age (years)	9.8 ± 1.7 (6.1–12.5)
BMI (kg/m^2^)	19.0 ± 4.1 (12.2–32.7)
*Category variable, n (%)*	
Gender	
Boys	149 (50.2)
Girls	148 (49.8)
*Annual family income (US$)* *^a^*	
≤$15,600	150 (50.5)
$15,600 – $31,250	101 (34.0)
≥ $31,250	44 (14.8)
Missing data	2 (0.7)
Father who used to work in the petrochemical complex	
Yes	145 (48.8)
No	152 (51.2)
Mother who used to work in the petrochemical complex	
Yes	58 (19.5)
No	239 (80.5)
*Physical activity* *^b^*	
Mild	105 (35.4)
Moderate	134 (45.1)
Vigorous	58 (19.5)
*Passive smoking*	
Yes	193 (65.0)
No	104 (35.0)

^a^US$ 1 = NT$ 32 (New Taiwan dollars).

^b^Mild physical activity including indoor sedentary activity (e.g., reading, using a computer, and cooking); moderate activity including taking a bus or train, walking, and shopping; and vigorous activity including outdoor exercise (playing ball, bicycling, and running).

### 3.2. Distributions of urinary trans, trans-muconic acid, and SPMA

The distribution of the urinary t,t-MA results are presented in Table 2. For urinary t,t-MA concentration, the geometric mean (GM) of the study participants from each school was as follows: school A, 12.18 ng/mL; school B, 15.52 ng/mL; school C, 10.45 ng/mL; school D, 7.48 ng/mL; school E, 5.28 ng/mL. The median urinary t,t-MA level of the study participants from each school was as follows: school A, 64.07 ng/mL; school B, 61.01 ng/mL; school C, 59.38 ng/mL; school D, 42.35 ng/mL; school E, ND (not detectable). The detection rate of urinary t,t-MA ranged from 48.5% (school E) to 70.6% (school B), with 0.3 ng/mL being the limit of detection for urinary t,t-MA. The results of the Kruskal–Wallis test did not reveal any significant difference in the median urinary t,t-MA levels of students in the five schools.

**Table 2:** Distributions of t,t-MA (ng/mL) and SPMA (ng/mL) in participants by different school groups (*N* = 297).

	*N*	% > LOD^a^		GM (GSD, 95%CI)	Min	Selected percentiles				Max	*p*-value^b^
						25th (95%CI)	50th (IQR, 95%CI)	75th (95%CI)	95th (95%CI)		
t,t-MA											0.662
	All	297	59.9	9.34 (32.47, 6.28–13.90)	ND	ND (ND-ND)	56.89 (144.76, 43.25–67.14)	144.91 (116.12–205.04)	578.69 (490.93–974.12)	21821.24	
	A	63	61.9	12.18 (37.45, 4.89–30.33)	ND	ND (ND-ND)	64.07 (220.45, ND-107.48)	220.60 (112.62–83.10)	754.74 (403.07–21821.24)	21821.24	
	B	51	70.6	15.52 (22.36, 6.48–37.18)	ND	ND (ND-45.72)	61.01 (102.04, 45.72–80.56)	102.19 (80.56–213.84)	522.53 (252.57–1039.69)	1039.69	
	C	63	60.3	10.45 (34.92, 4.27–25.56)	ND	ND (ND-ND)	59.38 (172.07, ND-96.17)	172.22 (100.54–421.95)	889.69 (460.23–1410.59)	1410.59	
	D	54	61.1	7.48 (24.62, 3.12–17.93)	ND	ND (ND-ND)	42.35 (102.76, ND-64.28)	102.91 (64.28–115.54)	276.90 (223.62–408.56)	408.56	
	E	66	48.5	5.28 (43.00, 2.09–13.31)	ND	ND (ND-ND)	ND (191.37, ND-120.66)	191.52 (123.13–295.77)	650.98 (430.76–4755.20)	4755.20	
SPMA											0.014^*^
	All	297	8.8	0.19 (2.17, 0.17–0.21)	ND	ND (ND-ND)	ND (0, ND-ND)	ND (ND-ND)	1.64 (1.09–2.34)	43.41	
	A	63	7.9	0.18 (2.07, 0.15–0.22)	ND	ND (ND-ND)	ND (0, ND-ND)	ND (ND-ND)	1.33 (ND-4.57)	4.57	
	B	51	2.0	0.16 (1.47, 0.14–0.18)	ND	ND (ND-ND)	ND (0, ND-ND)	ND (ND-ND)	ND (ND-2.34)	2.34	
	C	63	12.7	0.21 (2.39, 0.17–0.26)	ND	ND (ND-ND)	ND (0, ND-ND)	ND (ND-ND)	2.17 (1.13–3.37)	3.37	
	D	54	1.9	0.16 (1.35, 0.14–0.17)	ND	ND (ND-ND)	ND (0, ND-ND)	ND (ND-ND)	ND (ND-1.34)	1.34	
	E	66	16.7	0.24 (2.99, 0.18–0.31)	ND	ND (ND-ND)	ND (0, ND-ND)	ND (ND-1.06)	1.99 (1.48–43.41)	43.41	

a Limit of detection, ND was calculated as half of detection limit. The limit of detection for t,t-MA and SPMA were 0.3 and 0.3 ng/mL, respectively.

b Comparison of different school groups by Kruskal-Wallis test. *p < 0.05, **p < 0.01, ***p < 0.001. The bold value indicates the differences are significant.

The distribution of the urinary SPMA results are presented in Table 2. For urinary SPMA concentration, the GM of the study participants from each school was as follows: school A, 0.18 ng/mL; school B, 0.16 ng/mL; school C, 0.21 ng/mL; school D, 0.16 ng/mL; school E, 0.24 ng/mL. Median urinary SPMA levels were ND (A), ND (B), ND (C), ND (D), and ND (E), respectively. The detection rate for urinary SPMA ranged from 1.9% (school D) to 16.7% (school E), with 0.3 ng/mL being the limit of detection for SPMA. Significant differences in the median urinary SPMA levels of the five schools was identified through the Kruskal–Wallis test.

### 3.3. Distribution of estimated DI and HQ of benzene

The distribution of the estimated DI of benzene is presented in Table 3. The GM of the estimated benzene DI of the study participants from each school was as follows: school A, 2.96 μg/kg/day; school B, 4.16 μg/kg/day; school C, 2.39 μg/kg/day; school D, 2.01 μg/kg/day; school E, 1.38 μg/kg/day. The median (95th percentile, P_95_) estimated DI of benzene of the study participants from each school was as follows: school A, 11.13 (275.95) μg/kg/day; school B, 15.32 (95.04) μg/kg/day; school C, 14.81 (112.44) μg/kg/day; school D, 11.67 (40.87) μg/kg/day; school E, 0.14 (234.23) μg/kg/day. The results of the Kruskal–Wallis test did not reveal any significant difference in the estimated DI of benzene in the participants from the five schools.

**Table 3:** Distributions of estimated daily intake (μg/kg/day) for benzene in participants by different school groups (*N* = 297).

		*N*	GM (GSD, 95%CI)	Min	Selected percentiles				Max	*p*-value^a^
					25th (95%CI)	50th (IQR, 95%CI)	75th (95%CI)	95th (95%CI)		
Benzene										0.774
	All	297	2.36 (28.37, 1.61–3.46)	0.02	0.06 (0.05–0.07)	13.71 (35.41, 9.84–16.20)	35.47 (29.02–41.06)	130.52 (102.95–259.86)	4440.17	
	A	63	2.96 (32.54, 1.23–7.11)	0.02	0.06 (0.05–0.09)	11.13 (44.11, 0.14–17.91)	44.17 (19.31–93.09)	275.95 (118.61–4440.17)	4440.17	
	B	51	4.16 (19.61, 1.80–9.62)	0.03	0.07 (0.05–9.84)	15.32 (29.91, 9.84–19.46)	29.98 (19.46–55.96)	95.04 (74.24–185.88)	185.88	
	C	63	2.39 (30.24, 1.01–5.64)	0.02	0.06 (0.03–0.08)	14.81 (36.16, 0.10–18.75)	36.22 (22.32–66.95)	112.44 (71.56–491.67)	491.67	
	D	54	2.01 (19.77, 0.89–4.54)	0.02	0.07 (0.04–0.10)	11.67 (23.65, 0.10–16.91)	23.72 (16.91–30.67)	40.87 (30.96–75.67)	75.67	
	E	66	1.38 (40.08, 0.56–3.43)	0.02	0.05 (0.03–0.07)	0.14 (41.17, 0.07–31.45)	41.22 (31.92–60.80)	234.23 (91.21–1281.22)	1281.22	

a Comparison of different school groups by Kruskal-Wallis test. *p < 0.05, **p < 0.01, ***p < 0.001.

The distribution of the HQ of benzene is presented in Table 4. The median (P_95_) HQ of benzene with RfD proposed by the US EPA of the study participants from each school was as follows: school A, 2.78 (68.99); school B, 3.83 (23.76); school C, 3.70 (28.11); school D, 2.92 (10.22); school E, 0.03 (58.56). The median (P_95_) HQ of benzene with RfD proposed by ATSDR of the study participants from each school was as follows: school A, 22.26 (551.91); school B, 30.63 (190.08); school C, 29.63 (224.88); school D, 23.34 (81.73); school E, 0.27 (468.46). The median (P_95_) HQ of benzene with RfD proposed by Firth (24) of the study participants from each school was as follows: school A, 0.03 (0.69); school B, 0.04 (0.24); school C, 0.04 (0.28); school D, 0.03 (0.10); school E, 0.00 (0.59). We could see that more than half of the children have HQ higher than 1 when the RfDs proposed by the US EPA and ATSDR was applied.

**Table 4:** Distributions of hazard quotient for benzene in participants by different school groups (*N* = 297).

		*N*	% > 1^a^	GM (GSD, 95%CI)	Min	Selected percentiles				Max	*p*-value^b^
						25th (95%CI)	50th (IQR, 95%CI)	75th (95%CI)	95th (95%CI)		
HQ_US EPA_											0.774
	All	297	59.6	0.59 (28.37, 0.40–0.86)	<0.01	0.02 (0.01–0.02)	3.43 (8.85, 2.46–4.05)	8.87 (7.25–10.27)	32.63 (25.74–64.96)	1110.04	
	A	63	60.3	0.74 (32.54, 0.31–1.78)	<0.01	0.02 (0.01–0.02)	2.78 (11.02, 0.04–4.48)	11.04 (4.83–23.27)	68.99 (29.65–1110.04)	1110.04	
	B	51	70.6	1.04 (19.61, 0.45–2.40)	0.01	0.02 (0.01–2.46)	3.83 (7.48, 2.46–4.86)	7.50 (4.86–13.99)	23.76 (18.56–46.47)	46.47	
	C	63	60.3	0.60 (30.24, 0.25–1.41)	<0.01	0.01 (0.01–0.02)	3.70 (9.04, 0.03–4.69)	9.05 (5.58–16.74)	28.11 (17.89–122.92)	122.92	
	D	54	61.1	0.50 (19.77, 0.22–1.13)	0.01	0.02 (0.01–0.02)	2.92 (5.91, 0.02–4.23)	5.93 (4.23–7.67)	10.22 (7.74–18.92)	18.92	
	E	66	48.5	0.35 (40.08, 0.14–0.86)	<0.01	0.01 (0.01–0.02)	0.03 (10.30, 0.02–7.86)	10.31 (7.98–15.20)	58.56 (22.80–320.30)	320.30	
HQ_ATSDR_											0.774
	All	297	59.9	4.72 (28.37, 3.22–6.92)	0.03	0.12 (0.10–0.14)	27.41 (70.83, 19.69–32.40)	70.95 (58.04–82.13)	261.04 (205.91–519.71)	8880.34	
	A	63	61.9	5.92 (32.54, 2.46–14.23)	0.03	0.12 (0.09–0.18)	22.26 (88.23, 0.29–35.83)	88.35 (38.63–186.19)	551.91 (237.21–8880.34)	8880.34	
	B	51	70.6	8.33 (19.61, 3.61–19.23)	0.06	0.14 (0.09–19.69)	30.63 (59.83, 19.69–38.91)	59.97 (38.91–111.91)	190.08 (148.48–371.76)	371.76	
	C	63	60.3	4.78 (30.24, 2.03–11.28)	0.03	0.11 (0.05–0.16)	29.63 (72.32, 0.21–37.49)	72.43 (44.64–133.90)	224.88 (143.13–983.35)	983.35	
	D	54	61.1	4.02 (19.77, 1.78–9.07)	0.04	0.14 (0.08–0.19)	23.34 (47.29, 0.19–33.82)	47.43 (33.82–61.33)	81.73 (61.93–151.34)	151.34	
	E	66	48.5	2.77 (40.08, 1.12–6.86)	0.03	0.09 (0.05–0.13)	0.27 (82.36, 0.14–62.91)	82.45 (63.83–121.61)	468.46 (182.42–2562.43)	2562.43	
HQ_Firth_											0.774
	All	297	1.0	0.0059 (28.37, 0.0040–0.0086)	<0.0001	0.0002 (0.0001–0.0002)	0.0343 (0.0885, 0.0246–0.0405)	0.0887 (0.0725–0.1027)	0.3263 (0.2574–0.6496)	11.1004	
	A	63	1.6	0.0074 (32.54, 0.0031–0.0178)	<0.0001	0.0002 (0.0001–0.0002)	0.0278 (0.1102, 0.0004–0.0448)	0.1104 (0.0483–0.2327)	0.6899 (0.2965–11.1004)	11.1004	
	B	51	0	0.0104 (19.61, 0.0045–0.0240)	0.0001	0.0002 (0.0001–0.0246)	0.0383 (0.0748, 0.0246–0.0486)	0.0750 (0.0486–0.1399)	0.2376 (0.1856–0.4647)	0.4647	
	C	63	1.6	0.0060 (30.24, 0.0025–0.0141)	<0.0001	0.0001 (0.0001–0.0002)	0.0370 (0.0904, 0.0003–0.0469)	0.0905 (0.0558–0.1674)	0.2811 (0.1789–1.2292)	1.2292	
	D	54	0	0.0050 (19.77, 0.0022–0.0113)	0.0001	0.0002 (0.0001–0.0002)	0.0292 (0.0591, 0.0002–0.0423)	0.0593 (0.0423–0.0767)	0.1022 (0.0774–0.1892)	0.1892	
	E	66	1.5	0.0035 (40.08, 0.0014–0.0086)	<0.0001	0.0001 (0.0001–0.0002)	0.0003 (0.1030, 0.0002–0.0786)	0.1031 (0.0798–0.1520)	0.5856 (0.2280–3.2030)	3.2030	

a Reference doses (RfDs) of benzene by US EPA is 4 μg/kg/day, by ATSDR is 0.5 μg/kg/day, and by Firth (24) is 400 μg/kg/day.

b Comparison of different school groups by Kruskal–Wallis test. *p < 0.05, **p < 0.01, ***p < 0.001.

### 3.4. Comparison of urinary trans, trans-muconic acid concentrations, and benzene DI of participants from five schools

**Figure 2:**
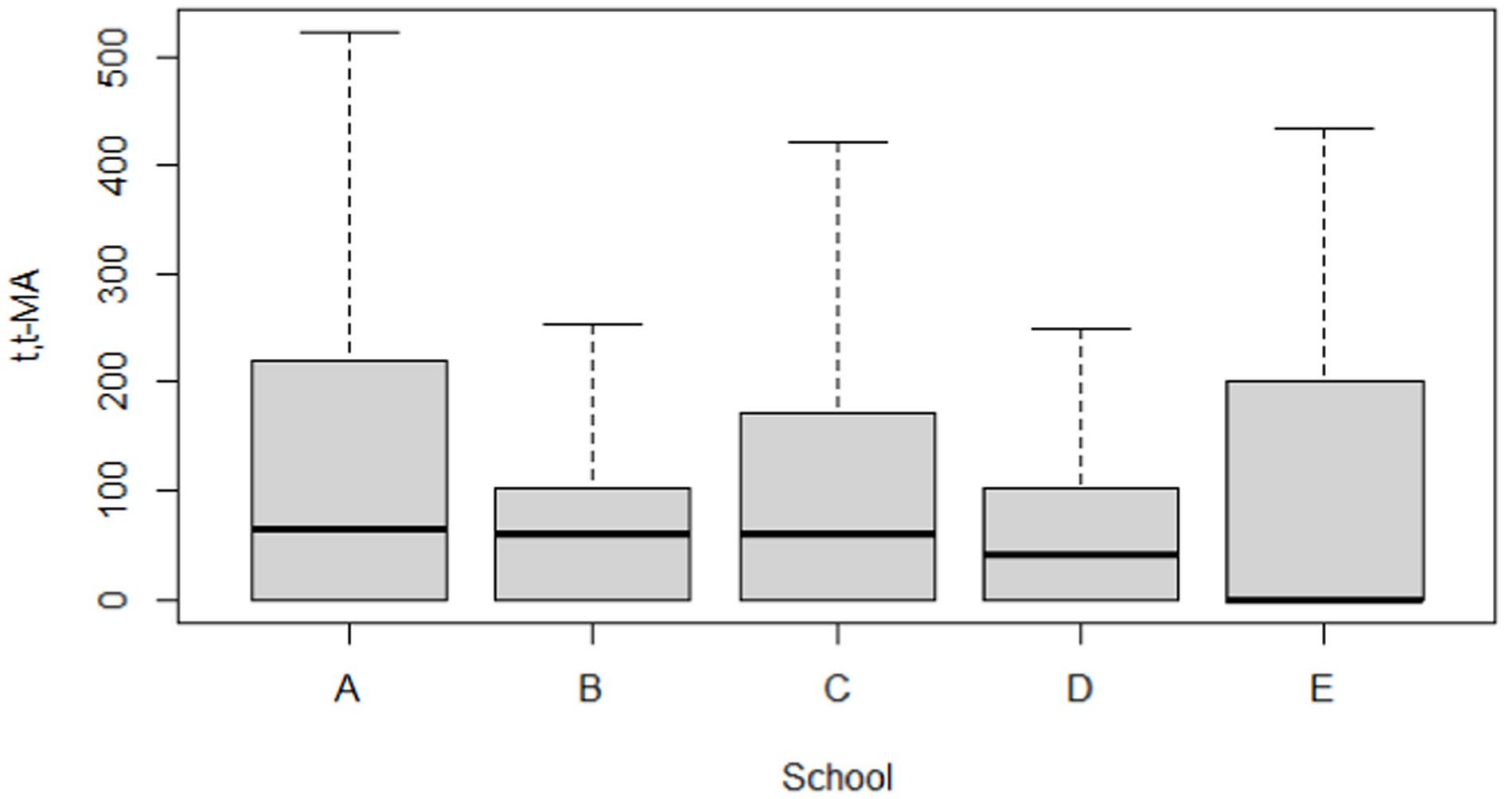
Boxplot of t,t-MA (ng/mL) in participants by different school groups (*N* = 297).

**Figure 3:**
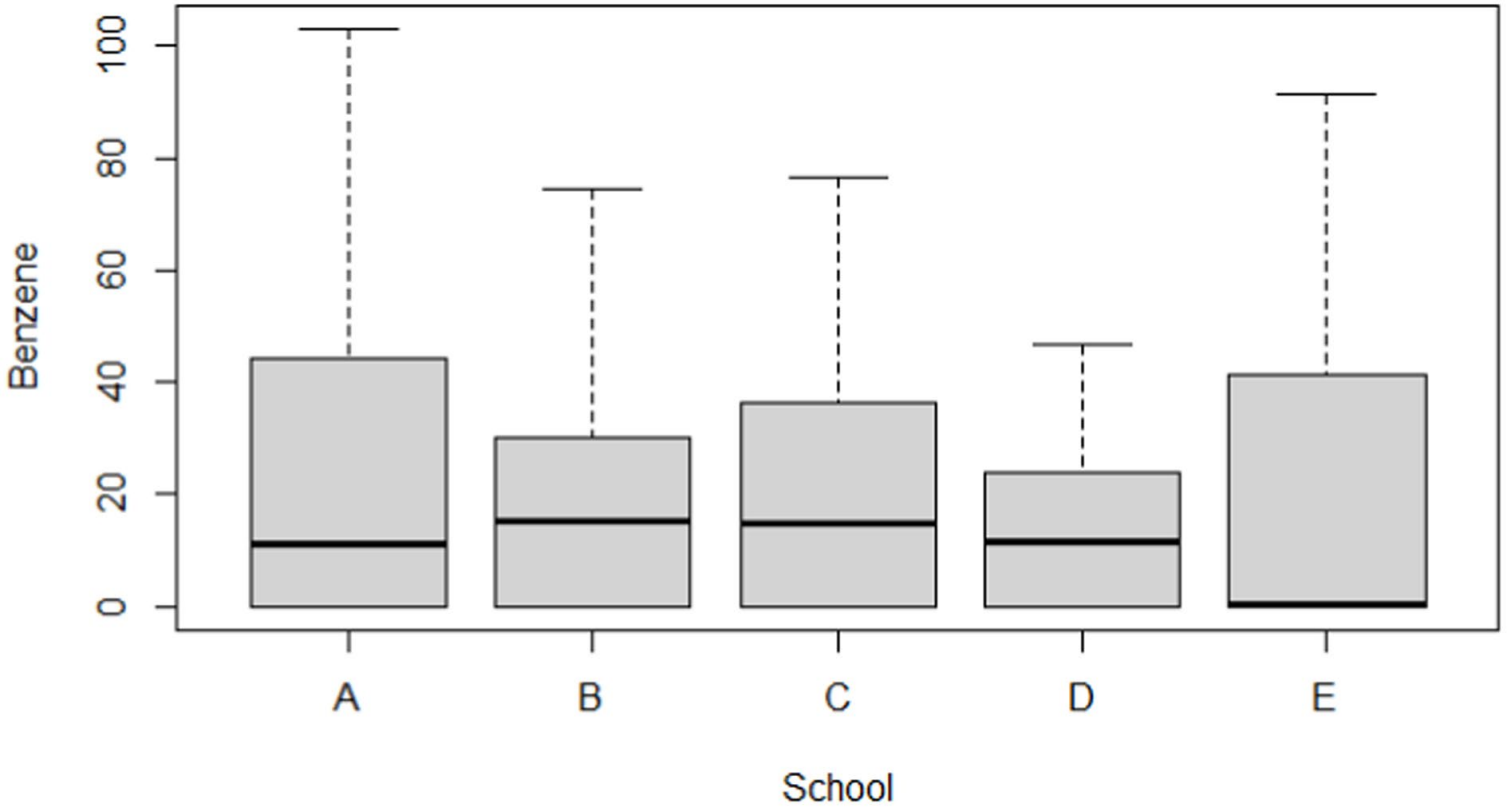
Boxplot of estimated daily intake (μg/kg/day) for benzene in participants by different school groups (*N* = 297).

Table 5 and Figures 2, 3 compare the urinary t,t-MA levels and estimated benzene DI of the participants from the five elementary schools. After ANCOVA adjustments were made for urinary creatinine, age, gender, passive smoking, BMI, having a father employed at a petrochemical complex, and close proximity of a student’s home to a main road, we could not identify a significant difference among the five schools for mean urinary t,t-MA level and estimated benzene DI; a Jonckheere–Terpstra test also did not reveal any significant difference with respect to these results.

**Table 5:** Comparison of urinary t,t-MA levels and estimated benzene daily intake for participants (*N* = 297) from different elementary schools after adjusting for covariance.

Scenario 1	A (*N* = 63)	B (*N* = 51)	C (*N* = 63)	D (*N* = 54)	E (*N* = 66)	*p*-value^a^	*p*-value^b^	*p*-trend^c^
Model^d^ (*n* = 297)								
Urinary t,t-MA (ng/mL)								0.248
Median	64.07	61.01	59.38	42.35	ND	0.662		
Mean	521.81	117.38	172.81	73.46	216.06		0.237	
Benzene daily intake (μg/kg/day)								0.295
Median	11.13	15.32	14.81	11.67	0.14	0.774		
Mean	109.85	27.03	35.82	14.53	53.48		0.267	

Scenario 2	A, B (*N* = 114)	C, D (*N* = 117)	E (*N* = 66)	*p*-value^a^	*p*-value^b^	*p*-trend^c^
Model^d^ (*n* = 297)						
Urinary t,t-MA (ng/mL)						0.368
Median	62.54	49.37	ND	0.627		
Mean	340.88	126.96	216.06		0.447	
Benzene daily intake (μg/kg/day)						0.310
Median	15.20	14.09	0.14	0.498		
Mean	72.80	25.99	53.48		0.522	

Scenario 3	A, B, C (*N* = 177)	D, E (*N* = 120)	*p*-value^a^	*p*-value^b^	*p*-trend^c^
Model^d^ (*n* = 297)					
Urinary t,t-MA (ng/mL)					0.164
Median	61.01	39.97	0.150		
Mean	281.06	151.89		0.401	
Benzene daily intake (μg/kg/day)					0.261
Median	15.02	10.60	0.261		
Mean	59.64	35.95		0.416	

a Kruskal–Wallis test for median.

b ANCOVA for mean.

c *p*-trend for Jonckheere–Terpstra Test.

d Model: ANCOVA adjusted for urinary creatinine, age, gender, passive smoking, BMI, father ever employed in the petrochemical complex, and home close to main road.

### 3.5. Multiple regression results

Table 6 presents the multiple regression results for the urinary t,t-MA levels of the participants from the five elementary schools after adjustments were made for covariates. We found that the creatinine-adjusted urinary t,t-MA- levels of the participants was marginally significantly and negatively associated with the distance between their school and the closest petrochemical complex after adjustments were made for urinary creatinine, gender, and age (school is located farther away from a nearby petrochemical complex: *β* = −0.26, 95% CI = −0.52 to 0.00, *p* = 0.053). In addition, the creatinine-adjusted urinary t,t-MA levels of the participants was significantly and negatively associated with their age (being older: β = −3.44, 95% CI = −5.46 to −1.41, *p* < 0.001). As for sensitivity analysis, we used only the children who (i) were not passively exposed to smoking and (ii) their fathers were not employed at the petrochemical complex as risk factors to assess the robustness of the reported associations. However, these two factors were all not significant after model adjusted for urinary creatinine (Supplementary Table S5).

**Table 6:** Multiple regression^a^ of urinary t,t-MA levels for the participating students attending 5 elementary schools in central Taiwan (*N* = 297).

Variables^b^	t,t-MA (*n* = 297)				
	*β*	SE	95% CI	*p*-value	*R* ^2^
Intercept	10.43	2.39	(5.72, 15.14)	<0.001^***^	0.047
Elementary school^c^	−0.26	0.13	(−0.52, 0.00)	0.053^#^	
Gender^c^	0.28	0.38	(−0.47, 1.03)	0.465	
Age (*y*)	−3.44	1.03	(−5.46, −1.41)	<0.001^***^	

a Multiple regression adjusted for urinary creatinine, gender, and age, ***p < 0.001, **p < 0.01, *p < 0.05, ^#^*p* < 0.1.

b Variables were natural logarithm transformed for t,t-MA, creatinine, and age.

c Dummy variables: we used school A as a reference and the order of other schools were increased by the increasing distance to the source; we also used girl as a reference of gender.

## 4. Discussion

In the present study, we assessed the urinary t,t-MA and SPMA levels of children from five elementary schools located close to VCM and PVC factories in central Taiwan. After adjustments were made for urinary creatinine, gender and age, the creatinine-adjusted urinary t,t-MA levels of the school-age children were revealed to be marginally significantly higher when the distance between their elementary school and a VCM or PVC factory was shorter.

Cigarette smoke was the main source of benzene exposure for smokers, whereas automobile exhaust or gasoline vapor emissions were the main sources for nonsmokers. Additionally, environmental tobacco smoke also contributed to people’s benzene exposure, with a small portion of the exposure originating from major point sources of benzene, such as petrochemical plants or refineries (6). Therefore, we compared the urinary t,t-MA and SPMA levels of the children in relation to several factors that could increase benzene exposure, and we discovered that the children who lived within 1 km of a VCM or PVC factory had a significantly higher level of benzene exposure (Supplementary Table S6).

The hazard quotient (HQ) refers to the ratio of the potential exposure to a substance to the level at which no adverse effects are expected. An HQ of >1 indicates potential adverse health effects. The HQ of our child participants with respect to benzene exposure was calculated on the basis of various RfDs. When the RfD proposed by the US EPA and ATSDR was applied, more than half of the children were at risk of adverse health effects caused by exposure to benzene (Table 4).

Human biomonitoring is a helpful method for differentiating environmental exposure by age, gender, region, and nationality; it can also generate foundational scientific evidence for developing public health policies. For urinary t,t-MA and SPMA, we compared the medians and P_95_ levels of populations in the United States (based on data retrieved from NHANES) and Canada (based on data retrieved from CHMS) and discovered that the benzene exposure level of our participants (aged 6–13 years) was close to those of similarly aged populations in the United States and Canada (Table 7). However, our participants were living in suburban area with low traffic, which would imply low benzene exposure from automobile exhaust or gasoline vapor emissions. The similar benzene exposure levels between our participants and similarly aged populations in the United States and Canada indicated that there could be other source of benzene exposure in our participants. Fustinoni et al. (25) reported that personal airborne exposure was a key risk factor for benzene exposure. Tsangari et al. (26) reported that participants living closer to the industrial cluster had higher benzene exposure. Therefore, living close to petrochemical complex could be the possible reason why our participants had high benzene exposure as similarly aged populations in the United States and Canada. Future research would be to determine the sources of benzene exposure from traffic, chemical transport vehicles, and industrial emissions in populations living near petrochemical complex.

**Table 7:** Comparison of urinary levels of t,t-MA (μg/L) and SPMA (μg/L) in general population among national surveys.

Country/Year	TPE3C, Taiwan^a^	NHANES, United States^b^	CHMS, Canada^c^	
	2013–2014	2017–2018	2012–2013	2014–2015
Age/ N	6–13	6–11	6–11	6–11
	297	252	496	508
t,t-MA	56.89 (578.69)	74.7 (1070)	55 (740)	61 (820)
SPMA	ND (1.64)	<LOD (0.447)	0.099 (0.41)	0.11 (0.49)

Data are presented as: median (P_95_); ND, not detected; LOD, limit of detection.

a This study.

b NHANES: National Report on Human Exposure to Environmental Chemicals.

c CHMS: Results of the Canadian Health Measures Survey Cycle 3 (2012–2013) and Cycle 3 (2014–2015).

We collected urine samples from the children to investigate their potential benzene exposure because the results from urine sample analysis can serve as an excellent indicator of recent exposure and the collection of urine samples is noninvasive. However, the present study has several limitations with respect to data interpretation. This was a cross-sectional study. The collected data only reflected the children’s exposure immediately before the survey. In addition, the collection of urine samples was conducted only once.

## 5. Conclusion

We concluded that most of the children who studied and lived close to a petrochemical complex may have been exposed to benzene. A follow-up study is required to further investigate their levels of benzene exposure and the potential health effects of such exposure.

## Data availability statement

The original contributions presented in the study are included in the article/Supplementary material, further inquiries can be directed to the corresponding author.

## Ethics statement

The studies involving humans were approved by Institutional Review Board of the National Health Research Institutes. The studies were conducted in accordance with the local legislation and institutional requirements. Written informed consent for participation in this study was provided by the participants’ legal guardians/next of kin.

## Author contributions

P-CH: conceptualization, methodology, software, writing – original draft, writing – review and editing, resources, and supervision. V-KP: methodology and writing – review and editing. KP: investigation and writing – review and editing. W-TC and H-IH: investigation and methodology. P-KC: investigation, methodology, and writing – original draft. All authors contributed to the article and approved the submitted version.

## Funding

This study was supported by Kaohsiung Municipal Siaogang Hospital, Kaohsiung Medical University, KMHK-H-109-007, KMHK-H-109-003, KMHK-H-110-009, KMHK-S-110-004, MOST 109-2314-B-037-141, MOST 110-2314-B-037-021, and NHRIKMU-111-I004-3. This work was supported partially by the Research Center for Precision Environmental Medicine, Kaohsiung Medical University, Kaohsiung, Taiwan from The Featured Areas Research Center Program within the framework of the Higher Education Sprout Project by the Ministry of Education (MOE) in Taiwan and by Kaohsiung Medical University Research Center Grant (KMU-TC111A01 and KMUTC111IFSP01). The authors thank the National Health Research Institutes for grants: EM-105-PP-16, EM-112-PP-11, NHRIKMU-111-I004-1, and EM-111-SP03.

## Conflict of interest

The authors declare that the research was conducted in the absence of any commercial or financial relationships that could be construed as a potential conflict of interest.

## Publisher’s note

All claims expressed in this article are solely those of the authors and do not necessarily represent those of their affiliated organizations, or those of the publisher, the editors and the reviewers. Any product that may be evaluated in this article, or claim that may be made by its manufacturer, is not guaranteed or endorsed by the publisher.
