# Supplementary material for: Exposure and risk assessment of urinary trans, trans-Muconic acid in school-age children in the vicinity of a petrochemical complex in Central Taiwan
Source: Front Public Health. 2023 Sep 4;11:1234823. doi: 10.3389/fpubh.2023.1234823 (PMC10507417; doi:10.3389/fpubh.2023.1234823)
Supplement: Supplementary file 1 [file Table_1.DOC]

Table S1. Distribution of TDGA (ng/mL) in participants by different school groups (N=297).

|  | **N** | **%>LOD ^a^** | **GM (GSD, 95%CI)** | **Min** | **Selected percentiles** | | | | **Max** | ***p*-value ^b^** |
| --- | --- | --- | --- | --- | --- | --- | --- | --- | --- | --- |
|  | | |  |  | **25^th^ (95%CI)** | **50^th^ (IQR, 95%CI)** | **75^th^ (95%CI)** | **95^th^ (95%CI)** |  |  |
| **TDGA** |  |  |  |  |  |  |  |  |  | **0.006^**^** |
| All | 297 | 90.9 | 223.78 (9.28, 173.51-288.61) | ND | 215.67 (187.73-243.83) | 356.34 (380.95, 317.77-396.82) | 596.62 (520.67-661.30) | 1311.34 (1028.57-2075.98) | 201628.96 |  |
| A | 63 | 93.7 | 319.37 (6.94, 196.09-520.15) | ND | 267.88 (157.79-332.49) | 472.46 (614.18, 338.18-583.04) | 882.06 (584.86-1134.88) | 1522.37 (1256.68-4574.30) | 4574.30 |  |
| B | 51 | 84.3 | 144.35 (16.21, 65.95-315.98) | ND | 199.18 (ND-292.96) | 344.89 (286.86, 292.96-403.01) | 486.04 (403.01-590.22) | 1126.90 (613.22-55146.59) | 55146.59 |  |
| C | 63 | 93.7 | 260.95 (6.46, 163.14-417.38) | ND | 227.50 (167.13-277.99) | 375.54 (396.02, 287.62-448.69) | 623.52 (472.48-698.83) | 914.58 (823.06-5096.83) | 5096.83 |  |
| D | 54 | 88.9 | 157.82 (10.76, 82.51-301.87) | ND | 177.53 (128.57-221.24) | 286.42 (226.66, 221.24-337.70) | 404.19 (337.70-471.34) | 615.65 (545.55-132661.13) | 132661.13 |  |
| E | 66 | 92.4 | 256.96 (8.79, 150.61-438.41) | ND | 217.31 (160.51-263.76) | 366.28 (473.53, 270.06-462.21) | 690.84 (489.49-839.98) | 1152.79 (880.62-201628.96) | 201628.96 |  |

^a^ Limit of detection, ND was calculated as half of detection limit. The limit of detection for TDGA was 0.7 ng/mL.

^b^ Comparison of different school groups by Kruskal-Wallis test. *p<0.05, **p<0.01, ***p<0.001.

Table S2. Distribution of creatinine (mg/dL) in participants by different school groups (N=297).

|  | **N** | **%>LOD ^a^** | **GM (GSD, 95%CI)** | **Min** | **Selected percentiles** | | | | **Max** | ***p*-value ^b^** |
| --- | --- | --- | --- | --- | --- | --- | --- | --- | --- | --- |
|  | | |  |  | **25^th^ (95%CI)** | **50^th^ (IQR, 95%CI)** | **75^th^ (95%CI)** | **95^th^ (95%CI)** |  |  |
| **Creatinine** |  |  |  |  |  |  |  |  |  | 0.343 |
| All | 297 | 100 | 96.65 (1.63, 91.44-102.17) | 30.50 | 65.50 (62.00-70.10) | 101.20 (75.4, 91.40-109.60) | 140.90 (131.70-151.70) | 195.72 (190.10-223.40) | 289.30 |  |
| A | 63 | 100 | 102.97 (1.66, 90.62-117.01) | 37.20 | 69.85 (58.00-82.60) | 112.40 (68.75, 85.30-126.00) | 138.60 (126.30-172.30) | 227.54 (195.80-289.30) | 289.30 |  |
| B | 51 | 100 | 92.42 (1.55, 81.63-104.64) | 41.40 | 63.55 (56.30-78.30) | 88.00 (61.45, 78.30-106.30) | 125.00 (106.30-165.50) | 186.85 (172.50-209.20) | 209.20 |  |
| C | 63 | 100 | 103.88 (1.60, 92.33-116.87) | 32.50 | 70.90 (60.80-84.90) | 115.60 (77.75, 85.60-137.10) | 148.65 (137.70-171.00) | 191.45 (180.20-202.50) | 202.50 |  |
| D | 54 | 100 | 91.63 (1.58, 80.83-103.87) | 32.60 | 65.40 (52.60-76.60) | 102.30 (57.00, 76.60-109.60) | 122.40 (109.60-150.00) | 174.04 (157.20-223.40) | 223.40 |  |
| E | 66 | 100 | 91.84 (1.70, 80.58-104.68) | 30.50 | 62.43 (48.30-73.80) | 95.50 (77.50, 74.10-112.10) | 139.93 (112.50-159.60) | 194.18 (174.00-256.00) | 256.00 |  |

^a^ Limit of detection, ND was calculated as half of detection limit. The limit of detection for creatinine was 10 mg/dL.

^b^ Comparison of different school groups by Kruskal-Wallis test. *p<0.05, **p<0.01, ***p<0.001.

Table S3. Distribution of smoothed creatinine excretion rate (mg/kg/day) in participants by different school groups (N=297).

|  | **N** | **GM (GSD, 95%CI)** | | **Min** | **Selected percentiles** | | | | **Max** | ***p*-value ^a^** |
| --- | --- | --- | --- | --- | --- | --- | --- | --- | --- | --- |
|  | | |  |  | **25^th^ (95%CI)** | **50^th^ (IQR, 95%CI)** | **75^th^ (95%CI)** | **95^th^ (95%CI)** |  |  |
| **CE_smoothed_** |  |  | |  |  |  |  |  |  | 0.511 |
| All | 297 | 17.34 (1.20, 16.98-17.70) | | 10.80 | 15.52 (15.05-15.94) | 17.65 (4.52, 17.04-18.05) | 20.04 (19.32-20.32) | 22.73 (21.62-23.63) | 26.27 |  |
| A | 63 | 17.76 (1.18, 17.05-18.50) | | 11.06 | 16.63 (15.05-16.94) | 18.08 (3.17, 16.99-18.77) | 19.80 (18.77-20.55) | 22.24 (20.81-25.63) | 25.63 |  |
| B | 51 | 17.60 (1.23, 16.61-18.66) | | 11.13 | 15.24 (13.41-16.14) | 17.92 (5.33, 16.14-19.26) | 20.57 (19.26-21.58) | 23.31 (21.71-26.27) | 26.27 |  |
| C | 63 | 16.87 (1.19, 16.15-17.61) | | 10.80 | 15.36 (13.75-15.99) | 17.05 (3.80, 16.11-17.74) | 19.16 (18.00-20.32) | 21.17 (20.48-23.63) | 23.63 |  |
| D | 54 | 17.47 (1.19, 16.64-18.33) | | 11.66 | 15.43 (13.81-16.32) | 17.88 (4.52, 16.32-18.83) | 19.95 (18.83-20.68) | 22.95 (20.85-23.99) | 23.99 |  |
| E | 66 | 17.09 (1.21, 16.29-17.92) | | 10.93 | 15.48 (14.23-16.21) | 17.03 (4.09, 16.29-18.28) | 19.57 (18.45-20.66) | 22.55 (21.00-25.96) | 25.96 |  |

^a^ Comparison of different school groups by Kruskal-Wallis test. *p<0.05, **p<0.01, ***p<0.001.

Table S4. Distribution of weight (kg) in participants by different school groups (N=297).

|  | **N** | **GM (GSD, 95%CI)** | | **Min** | **Selected percentiles** | | | | **Max** | ***p*-value ^a^** |
| --- | --- | --- | --- | --- | --- | --- | --- | --- | --- | --- |
|  | | |  |  | **25^th^ (95%CI)** | **50^th^ (IQR, 95%CI)** | **75^th^ (95%CI)** | **95^th^ (95%CI)** |  |  |
| **Weight** |  |  | |  |  |  |  |  |  | **0.026 ^*^** |
| All | 297 | 35.49 (1.37, 34.25-36.79) | | 18.70 | 28.40 (27.10-29.50) | 33.90 (14.40, 32.65-35.40) | 42.80 (41.00-46.90) | 65.18 (58.50-67.90) | 84.70 |  |
| A | 63 | 33.10 (1.35, 30.67-35.72) | | 18.70 | 26.68 (24.30-29.50) | 31.70 (10.75, 29.50-33.90) | 37.43 (33.95-46.55) | 61.78 (50.10-74.20) | 74.20 |  |
| B | 51 | 34.82 (1.41, 31.62-38.35) | | 19.90 | 26.55 (23.50-29.00) | 34.30 (19.65, 29.00-37.30) | 46.20 (37.30-52.60) | 63.40 (52.90-71.50) | 71.50 |  |
| C | 63 | 39.53 (1.39, 36.38-42.95) | | 19.70 | 32.00 (27.20-33.50) | 37.50 (18.05, 33.70-42.50) | 50.05 (42.70-57.00) | 69.07 (64.20-84.70) | 84.70 |  |
| D | 54 | 35.28 (1.30, 32.87-37.87) | | 22.50 | 28.80 (27.80-30.80) | 33.60 (13.70, 30.80-36.50) | 42.50 (36.50-45.10) | 57.32 (48.10-76.60) | 76.60 |  |
| E | 66 | 34.91 (1.36, 32.38-37.65) | | 18.80 | 28.70 (26.00-30.40) | 34.50 (12.70, 30.50-37.20) | 41.40 (37.30-47.60) | 65.05 (50.40-72.00) | 72.00 |  |

^a^ Comparison of different school groups by Kruskal-Wallis test. *p<0.05, **p<0.01, ***p<0.001.

Table S5. Sensitivity analysis: multiple regression^a^ of urinary t,t-MA levels for the participating students attending 5 elementary schools in central Taiwan (N=297).

| **Variables^b^** | **t,t-MA (n=297)** | | | | |
| --- | --- | --- | --- | --- | --- |
|  | *β* | SE | 95% CI | *P* value | *R^2^* |
| Intercept | -8.44 | 1.82 | (-12.01, -4.86) | <0.001 ^***^ | 0.106 |
| Passive smoking^c^ | 0.18 | 0.40 | (-0.62, 0.97) | 0.658 |  |
| Creatinine | 2.31 | 0.40 | (1.53, 3.09) | <0.001 ^***^ |  |
| **Variables^b^** | **t,t-MA (n=297)** | | | | |
|  | *β* | SE | 95% CI | *P* value | *R^2^* |
| Intercept | -8.43 | 1.82 | (-12.01, -4.85) | <0.001 ^***^ | 0.106 |
| Father ever employed by a petrochemical complex^c^ | 0.09 | 0.38 | (-0.67, 0.84) | 0.403 |  |
| Creatinine | 2.32 | 0.40 | (1.54, 3.10) | <0.001 ^***^ |  |

^a^ Multiple regression adjusted for urinary creatinine and passive smoking or father ever employed in the petrochemical complex, ****p*<0.001, ***p*<0.01, **p*<0.05, ^#^*p*<0.1.

^b^ Variables were logarithm transformed for t,t-MA and creatinine.

^c^ Dummy variables: we used non-smoker and never served in petrochemical complex as a reference of passive smoking and father's job, respectively.

Table S6. Comparison of urinary t,t-MA and SPMA levels (ng/mL) for several factors a

| t,t-MA (median) | Incense use | Mosquito coil use | Passive smoking | Home close to main road | Factories within 1 km near living environment |
| --- | --- | --- | --- | --- | --- |
| Yes | 55.53 | 59.85 | 59.16 | 56.59 | 85.15 * |
| No | 57.21 | 51.05 | 45.43 | 57.89 | 46.27 |
| SPMA (median) |  |  |  |  |  |
| Yes | ND | ND | ND | ND | ND |
| No | ND | ND | ND | ND | ND |

^a^ Comparison of different groups by Mann-Whitney U test. *p<0.05, **p<0.01, ***p<0.001.
